# Supplementary material for: Chlamydia evasion of neutrophil host defense results in NLRP3 dependent myeloid-mediated sterile inflammation through the purinergic P2X7 receptor
Source: Nat Commun. 2021 Sep 15;12:5454. doi: 10.1038/s41467-021-25749-3 (PMC8443728; doi:10.1038/s41467-021-25749-3)
Supplement: Supplementary file 1 — Supplementary Information [file 41467_2021_25749_MOESM1_ESM.pdf]

Chlamydia evasion of neutrophil host defense results in NLRP3 dependent myeloid-mediated sterile inflammation through the purinergic P2X7 receptor

Chunfu Yang, Lei Lei, John W. Marshall Collins, Michael Briones, Li Ma, Gail L. Sturdevant, Hua Su, Anuj K. Kashyap, Dave Dorward, Kevin W. Bock, Ian N. Moore, Christine Bonner, Chih-Yu Chen, Craig Martens, Stacy Ricklefs, Masahiro Yamamoto, Kiyoshi Takeda, Yoichiro Iwakura, Grant McClarty and Harlan D. Caldwell*

SUPPLEMENTARY INFORMATION

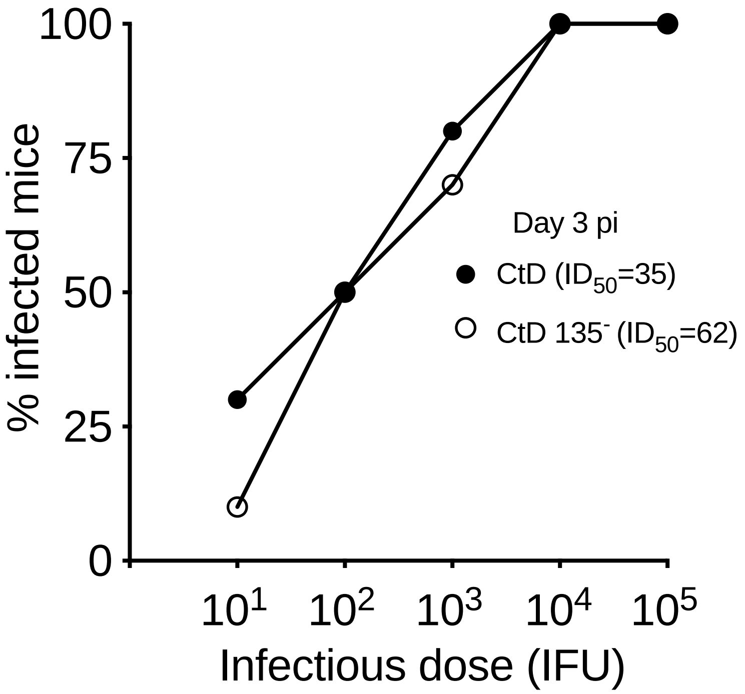

Supplementary Figure 1. **ID_{50} of CtD and CtD $CT135^-$ in the female mouse genital tract.** C57BL/6 mice (n=10 mice per group) were infected transcervically with CtD or CtD $CT135^-$. Recoverable IFU in vaginal swabs were assayed at day 3 pi. The ID_{50} of CtD and CtD $CT135^-$ strains at day 3 are calculated by logit analysis.

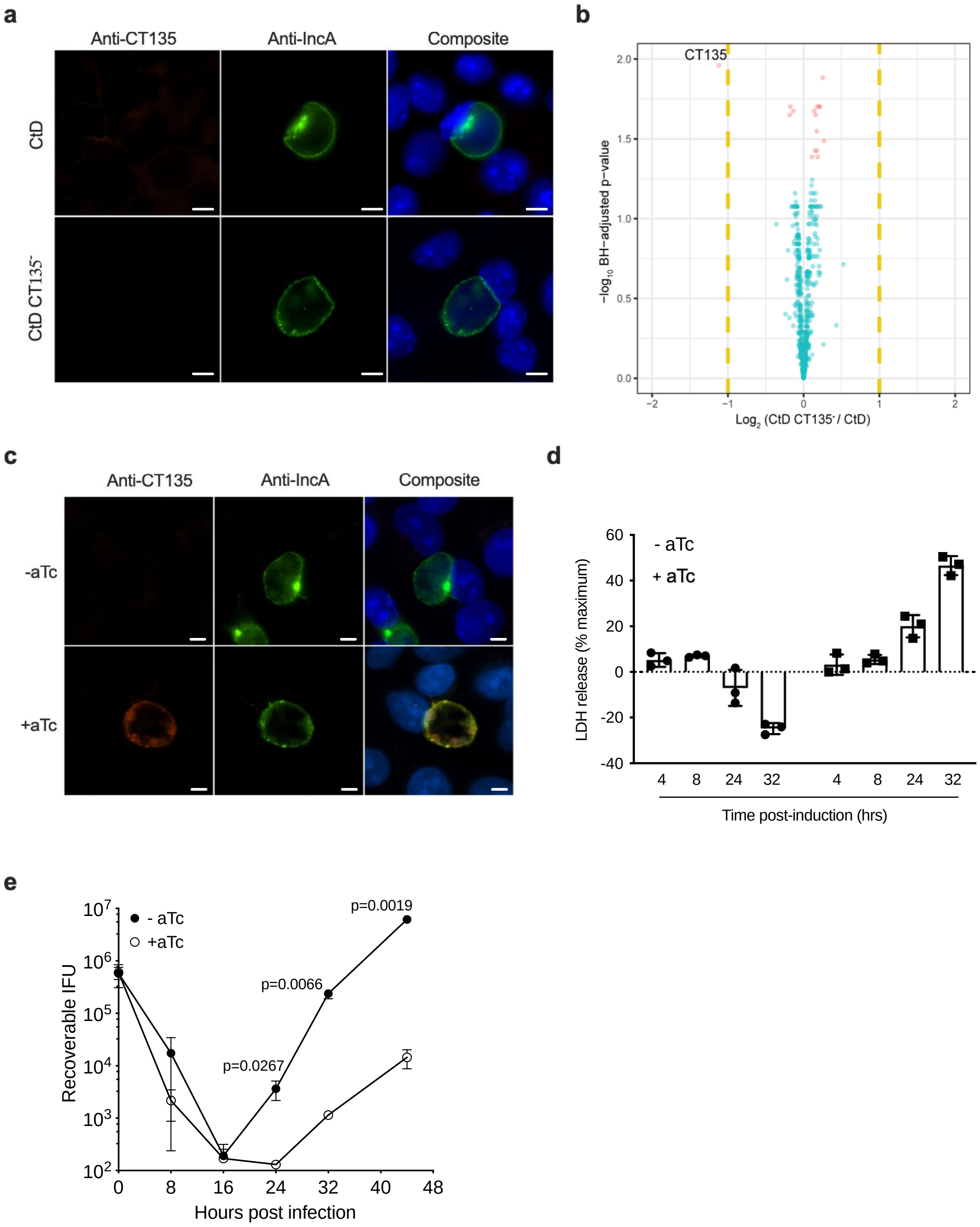
a
Anti-CT135
Anti-IncA
Composite
CtD
CtD CT135⁻
b
CT135
−log10 BH−adjusted p−value
Log2 (CtD CT135⁻/ CtD)
c
Anti-CT135
Anti-IncA
Composite
-aTc
+aTc
d
- aTc
+ aTc
LDH release (% maximum)
Time post-induction (hrs)
e
- aTc
+aTc
p=0.0019
p=0.0066
p=0.0267
Recoverable IFU
Hours post infection

Supplementary Figure 2. **Phenotype of CtD, CtD CT135$^-$ and complement strain CtD CT135$^-$ *::bla* CT135.** **a**, Immunofluorescence of CtD and CtD CT135$^-$ infected HeLa 229 cells at 40 hrs pi stained with anti-CT135 (RED), anti-IncA (Green) and DAPI (blue). **b**, Expression of CT135 by CtD and CtD CT135$^-$ strains by proteomic analysis. Volcano plots detect CT135 protein only in CtD infected HeLa cells. **c**, IFA of HeLa cells infected with CtD CT135$^-$ *::bla* CT135. Anhydrotetracycline hydrochloride (aTC) (50 ng/ml) was added for 8 hrs to induce the expression of CT135. Infected cells were fixed with cold methanol and stained with anti-CT135 (RED), anti-IncA (Green) and DAPI (blue). **d**, HeLa cells infected with CtD CT135$^-$ *::bla* CT135, induced with aTC and then assayed for LDH released at various times post induction. **e,** aTC induced CtD CT135$^-$*::bla* CT135 exhibit significantly reduced infectious progeny in HeLa 229 cells compared to uninduced CtD CT135$^-$*::bla* CT135. One-step growth curves were performed in HeLa 229 cells. HeLa 229 cells were infected at a MOI of 0.5. Cells were harvested at different times post-infection and the number of rIFU were determined (n = 3, two independent experiments were performed, two-tailed unpaired student's t tests). **d-e** Data are shown as the mean ± SD. **a** and **c** Images shown are representative of two independent experiments. Scale bar, 10 µm.

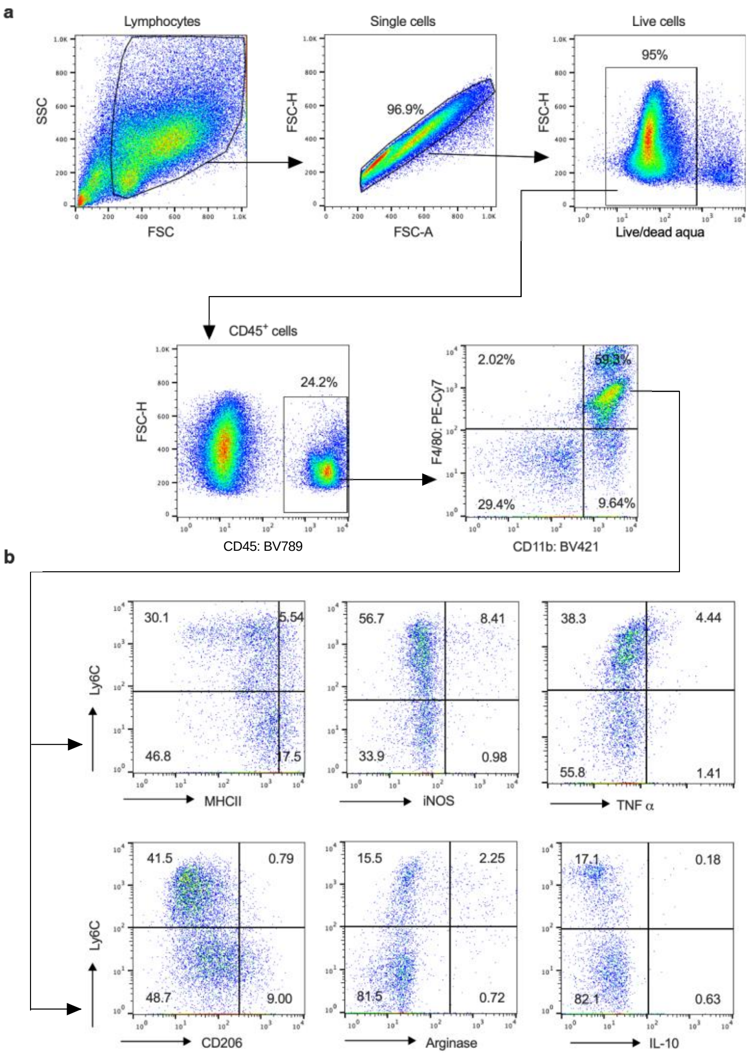

Supplementary Figure 3. **Flow cytometry gating strategies.** Example of the gating strategy for GT macrophage cell phenotyping by flow cytometry. **a,** Gate strategy to sort CD45$^+$ CD11b$^+$ F4/80$^+$ cells for Figs. **1f, 2e and 6g**. **b,** Gate strategy to sort CD45$^+$ CD11b$^+$ F4/80$^+$ Ly6C$^+$ MHCII$^+$, CD45$^+$ CD11b$^+$ F4/80$^+$ Ly6C$^+$ iNOS$^+$, CD45$^+$ CD11b$^+$ F4/80$^+$ Ly6C$^+$ TNFα^+, CD45$^+$ CD11b$^+$ F4/80$^+$ Ly6C$^+$ CD206$^+$ cells, CD45$^+$ CD11b$^+$ F4/80$^+$ Ly6C$^+$ Arginase$^+$ cells and CD45$^+$ CD11b$^+$ F4/80$^+$ Ly6C$^+$ IL10$^+$ cells presented on Figs. **1h, 2g and 6i** and Supplementary Figs. **4a-e, 5a-e and 8a-e.**

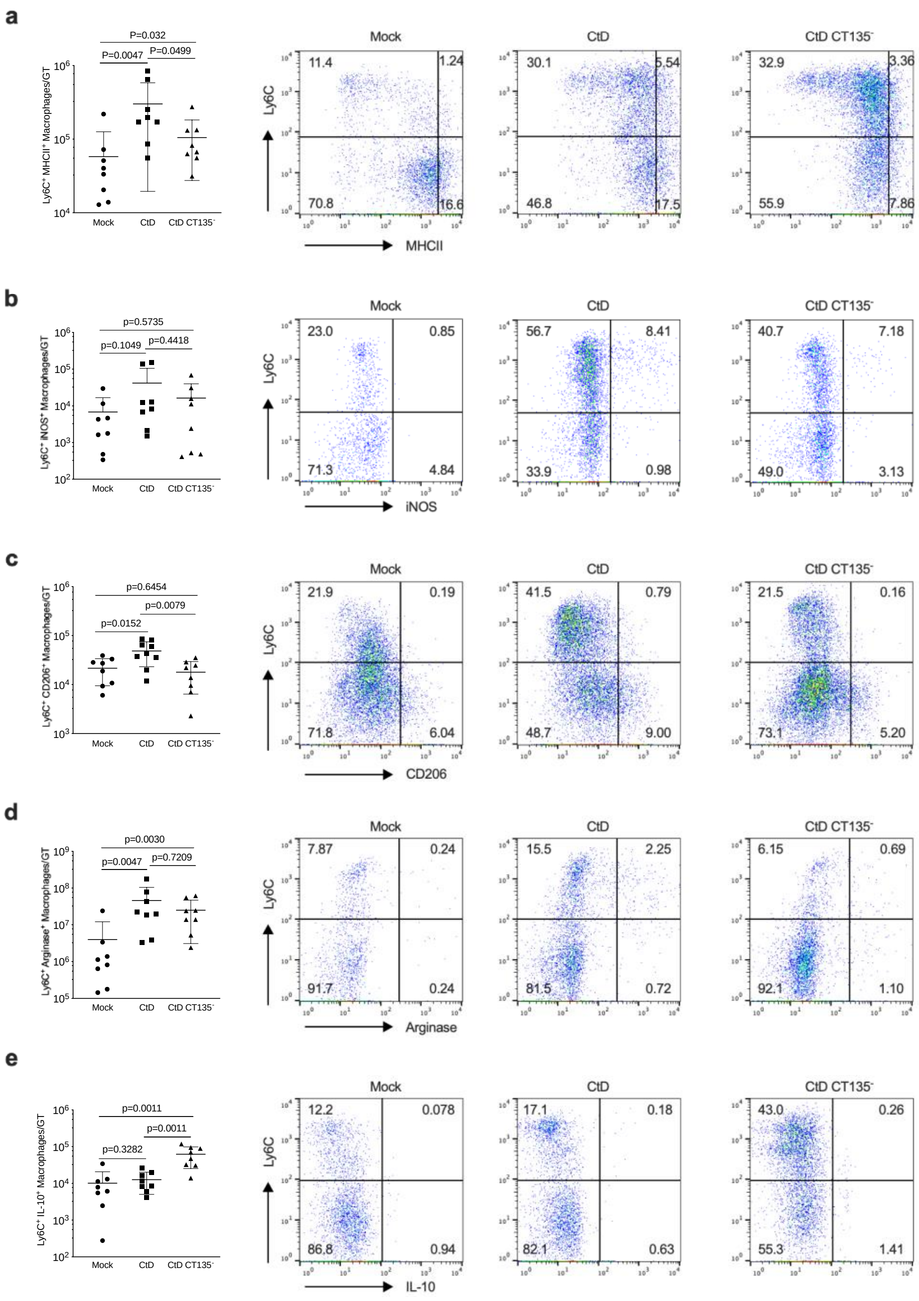

Supplementary Figure 4. **Flow cytometry of CD11b$^+$ F4/80$^+$ Ly6C$^+$ macrophages in entire GT of mock, CtD, and CtD CT135$^-$ infected C57BL/6 mice at day 56 pi.** **a**, Ly6C$^+$ MHC II$^+$ macrophages. **b**, Ly6C$^+$ iNOS$^+$ macrophages. **c**, Ly6C$^+$ CD206$^+$ macrophages. **d**, Ly6C$^+$ Arginase$^+$ macrophages. **e**, Ly6C$^+$ IL-10$^+$ macrophages. (**a-e,** n=8 mice per group, pool from two independent experiments, two-tailed Mann–Whitney test). **a-e** Data are shown as the mean ± SD.

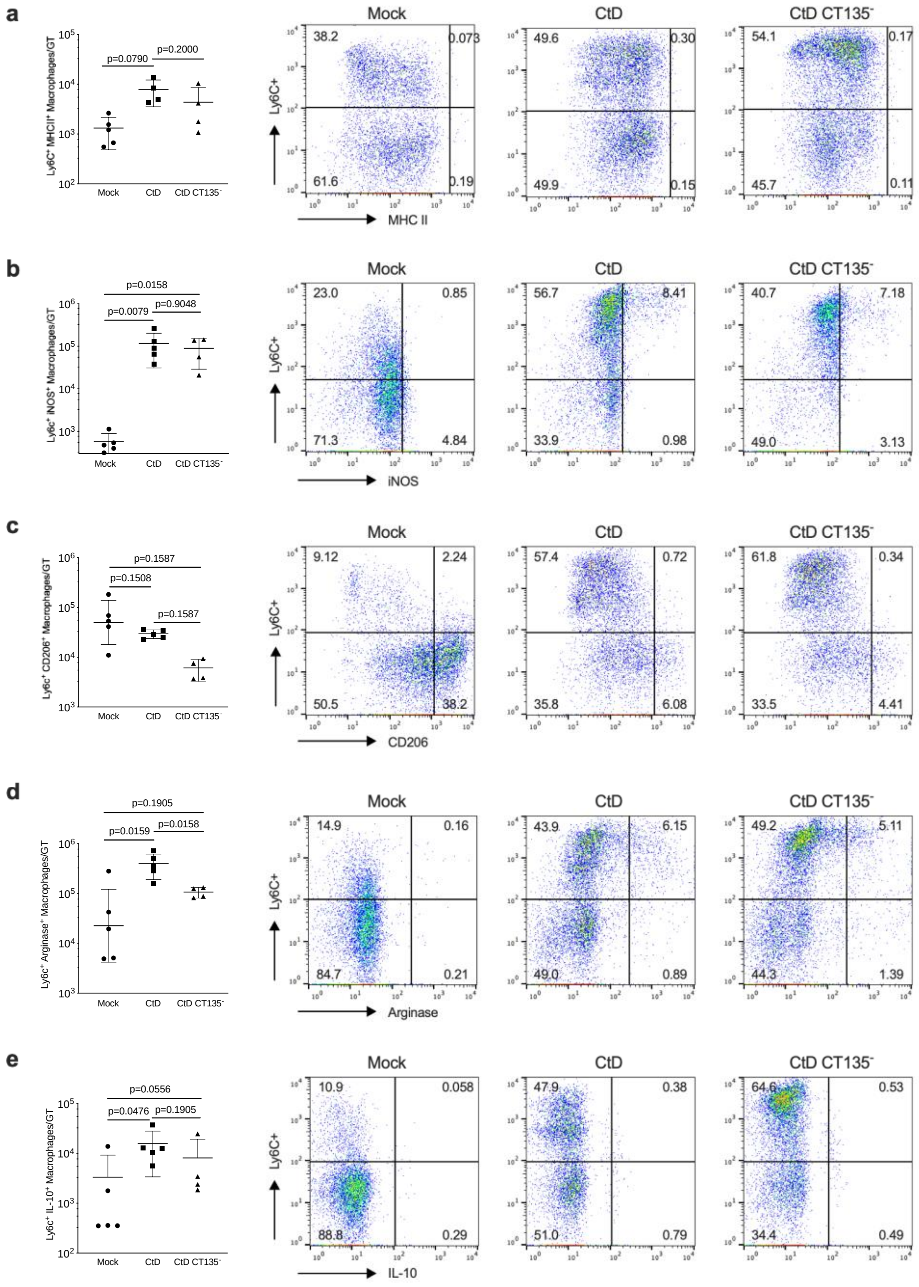

Supplementary Figure 5. **Flow cytometry of CD11b^{+} F4/80^{+} Ly6C^{+} macrophages in entire GT of mock, CtD, and CtD CT135^{-} infected RAG2$^{-/-}$ mice at day 56 pi.** **a**, Ly6C^{+} MHC II^{+} macrophages. **b**, Ly6C^{+} iNOS^{+} macrophages. **c**, Ly6C^{+} CD206^{+} macrophages. **d**, Ly6C^{+} Arginase^{+} macrophages. **e**, Ly6C^{+} IL-10^{+} macrophages. (**a-e,** n=5 mice per group, two-tailed Mann–Whitney test). **a-e** Data are shown as the mean ± SD.

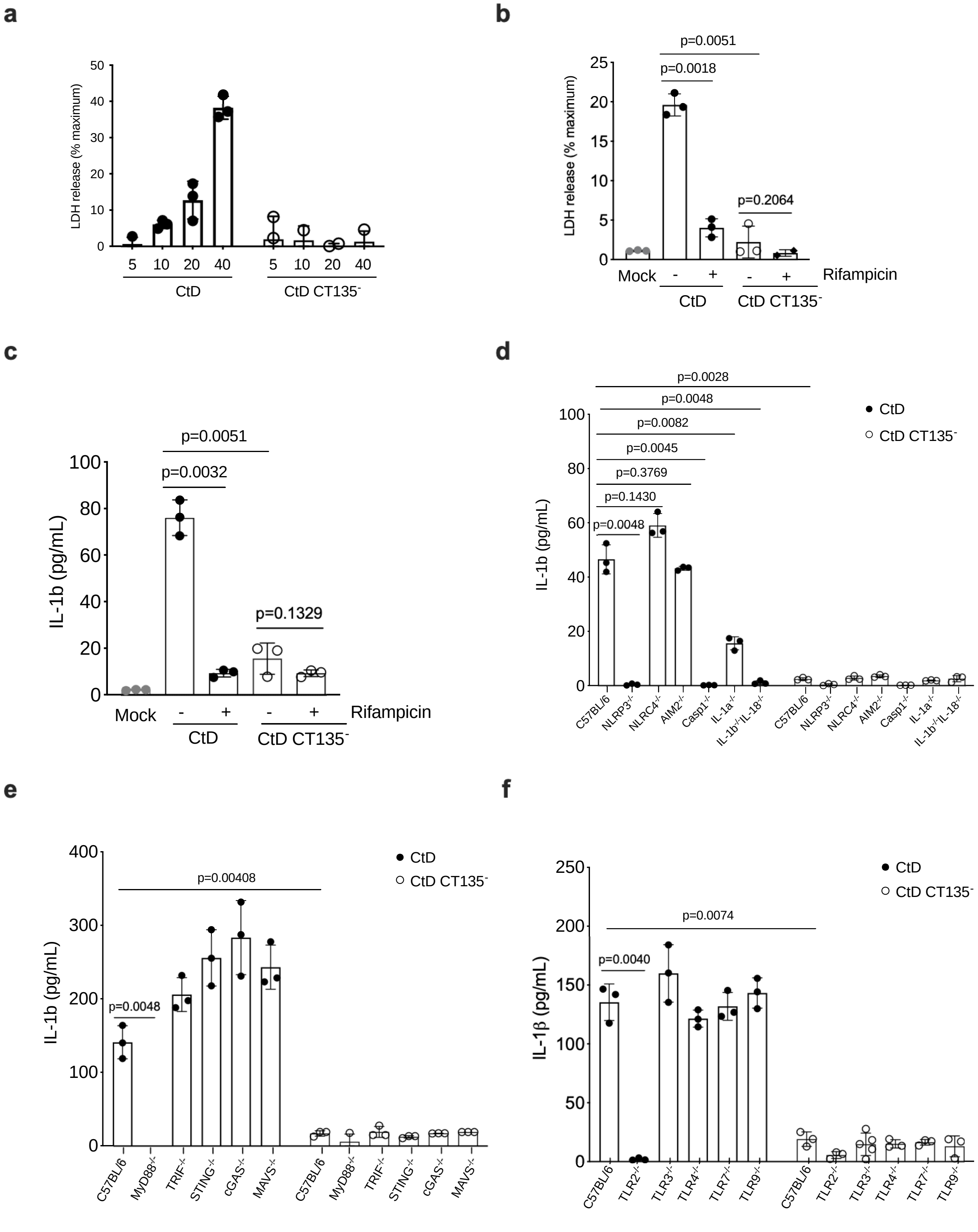
a
LDH release (% maximum)
5 10 20 40 5 10 20 40
CtD
CtD CT135-
b
LDH release (% maximum)
p=0.0051
p=0.0018
p=0.2064
Mock - + - + Rifampicin
CtD
CtD CT135-
c
IL-1b (pg/mL)
p=0.0051
p=0.0032
p=0.1329
Mock - + - + Rifampicin
CtD
CtD CT135-
d
IL-1b (pg/mL)
p=0.0028
p=0.0048
p=0.0082
p=0.0045
p=0.3769
p=0.1430
p=0.0048
CtD
CtD CT135-
C57BL/6 NLRP3-/- NLRC4-/- AIM2-/- Casp1-/- IL-1a-/- IL-1b-/-IL-18-/- C57BL/6 NLRP3-/- NLRC4-/- AIM2-/- Casp1-/- IL-1a-/- IL-1b-/-IL-18-/-
e
IL-1b (pg/mL)
p=0.00408
p=0.0048
CtD
CtD CT135-
C57BL/6 MyD88-/- TRIF-/- STING-/- cGAS-/- MAVS-/- C57BL/6 MyD88-/- TRIF-/- STING-/- cGAS-/- MAVS-/-
f
IL-1β (pg/mL)
p=0.0074
p=0.0040
CtD
CtD CT135-
C57BL/6 TLR2-/- TLR3-/- TLR4-/- TLR7-/- TLR9-/- C57BL/6 TLR2-/- TLR3-/- TLR4-/- TLR7-/- TLR9-/-

Supplementary Figure 6. **Infection of BMDM with CtD and CtD CT135^{-} strains.** **a**, LDH release by bone marrow derived macrophage (BMDM) infected with different MOI of CtD and CtD CT135^{-}. **b**, CT135 dependent LDH release in BMDM is dependent on chlamydial *de novo* mRNA synthesis. (Two-tailed unpaired student's t tests). **c**, IL-1β secretion of BMDM infected with CtD and CtD CT135^{-}. (Two-tailed Mann–Whitney test). **d**, Secretion of IL-1β in CtD and CtD CT135^{-} infected BMDM derived from C57BL/6, NLRP3$^{-/-}$, NLRC4$^{-/-}$, AIM2$^{-/-}$, Casp1$^{-/-}$, IL-1α$^{-/-}$, IL-1β$^{-/-}$IL18$^{-/-}$ mice. **e**. Secretion of IL-1β in CtD and CtD CT135^{-}infected BMDM derived from C57BL/6, MyD88$^{-/-}$, TRIF$^{-/-}$, STING$^{-/-}$, cGAS$^{-/-}$, MAVS$^{-/-}$ mice**.** **f**, Secretion of IL-1β in CtD and CtD CT135^{-}infected BMDM derived from C57BL/6, TLR2$^{-/-}$, TLR3$^{-/-}$, TLR4$^{-/-}$, TLR7$^{-/-}$, TLR9$^{-/-}$ mice. **d-f** One-tailed unpaired student's t tests. a-f, n=3 mice per group, two independent experiments were performed. **a-f** Data are shown as the mean ± SD.

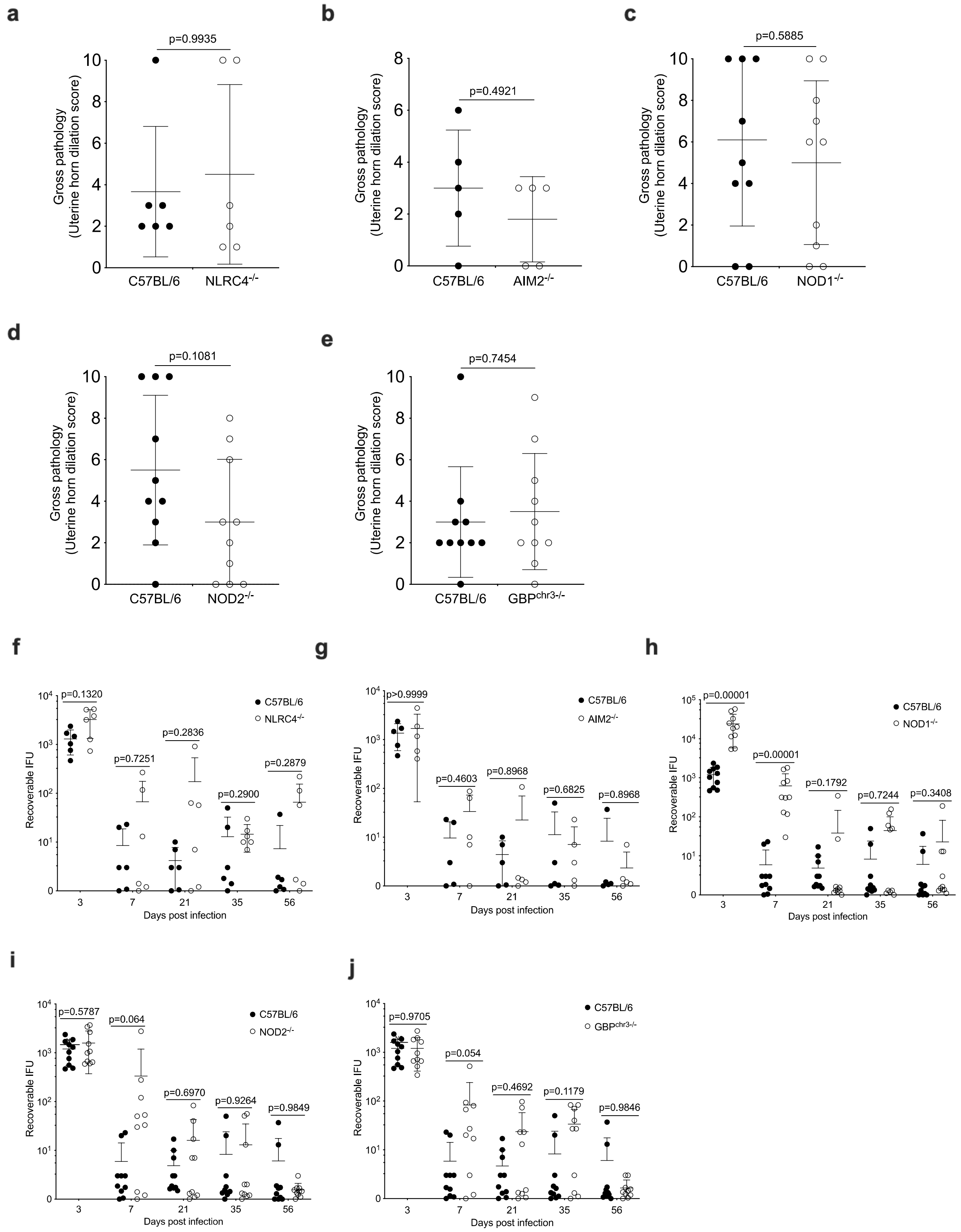
a
p=0.9935
Gross pathology (Uterine horn dilation score)
C57BL/6
NLRC4-/-
b
p=0.4921
Gross pathology (Uterine horn dilation score)
C57BL/6
AIM2-/-
c
p=0.5885
Gross pathology (Uterine horn dilation score)
C57BL/6
NOD1-/-
d
p=0.1081
Gross pathology (Uterine horn dilation score)
C57BL/6
NOD2-/-
e
p=0.7454
Gross pathology (Uterine horn dilation score)
C57BL/6
GBPchr3-/-
f
p=0.1320
p=0.7251
p=0.2836
p=0.2900
p=0.2879
Recoverable IFU
Days post infection
C57BL/6
NLRC4-/-
g
p>0.9999
p=0.4603
p=0.8968
p=0.6825
p=0.8968
Recoverable IFU
Days post infection
C57BL/6
AIM2-/-
h
p=0.00001
p=0.00001
p=0.1792
p=0.7244
p=0.3408
Recoverable IFU
Days post infection
C57BL/6
NOD1-/-
i
p=0.5787
p=0.064
p=0.6970
p=0.9264
p=0.9849
Recoverable IFU
Days post infection
C57BL/6
NOD2-/-
j
p=0.9705
p=0.054
p=0.4692
p=0.1179
p=0.9846
Recoverable IFU
Days post infection
C57BL/6
GBPchr3-/-

Supplementary Figure 7. **Infection and gross pathology of CtD infected C57BL/6 and inflammasome associated KO mice at day 56 pi.** **a-f**, Quantification of gross uterine horn pathology. **a**, C57BL/6 and NLRC4$^{-/-}$ mice (n=6 mice per group). **b**, C57BL/6 and AIM2$^{-/-}$ mice (n=6 mice per group). **c**, C57BL/6 and NOD1$^{-/-}$ mice (n=10 mice per group). **d**, C57BL/6 and NOD2$^{-/-}$ mice (n=10 mice per group). **e**, C57BL/6 and GBP$^{chr3-/-}$ mice (n=10 mice per group). (**a-e,** two-tailed Mann–Whitney test). **f-k**, Infection kinetics. **f**, C57BL/6 and NLRC4$^{-/-}$ mice (n=6 mice per group). **g**, C57BL/6 and AIM2$^{-/-}$ mice (n=6 mice per group). **h**, C57BL/6 and NOD1$^{-/-}$ mice (n=10 mice per group). **i**, C57BL/6 and NOD2$^{-/-}$ mice (n=10 mice per group). **j**. C57BL/6 and GBP$^{chr3-/-}$ mice (n=10 mice per group). (**f-k,** two-tailed Mann–Whitney test). **a-j** Data are shown as the mean ± SD. **a-j** Data are pooled from two independent experiments.

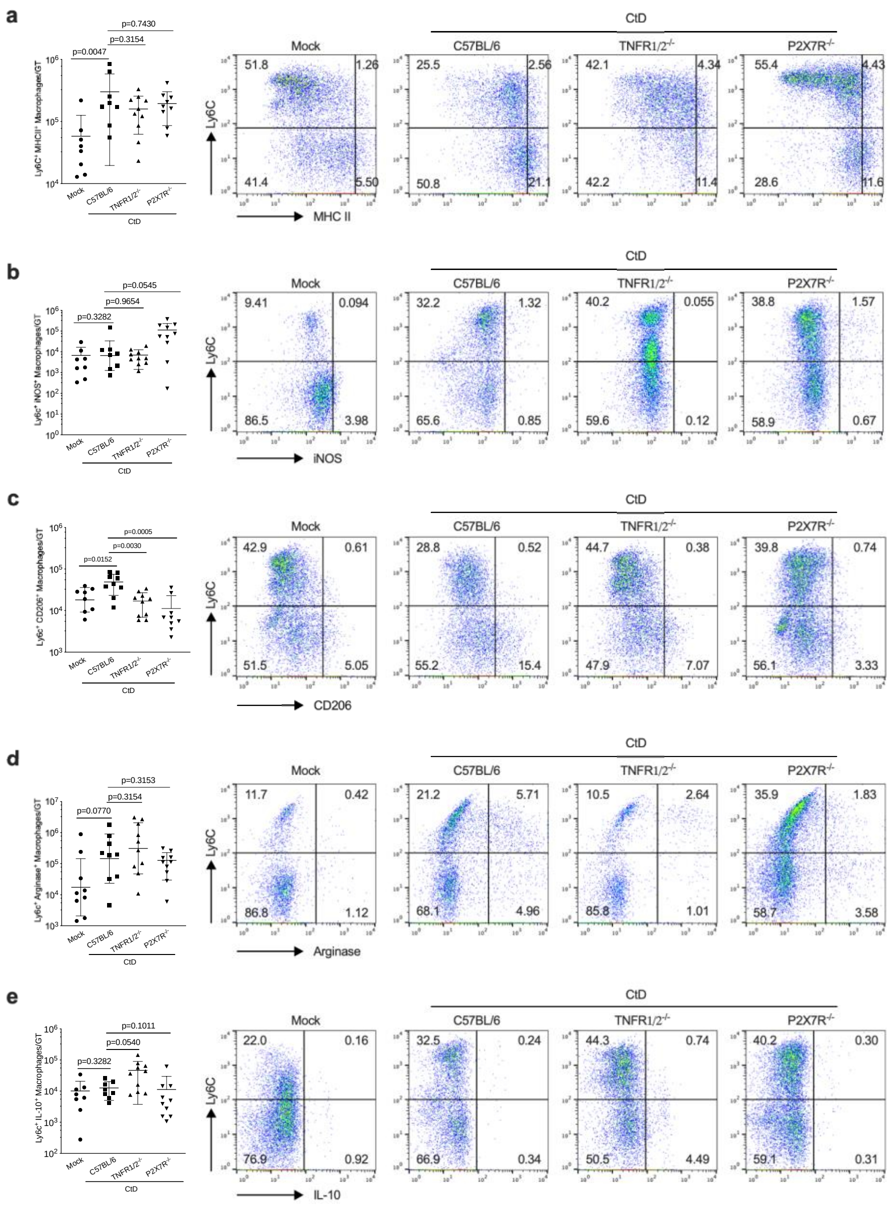

Supplementary Figure 8. **Flow cytometry of CD11b$^+$ F4/80$^+$ Ly6C$^+$ macrophages in entire GT of mock and CtD infected C57BL/6, P2X7R$^{-/-}$ and TNFR1/2$^{-/-}$ mice at day 56 pi.** **a**, Ly6C$^+$ MHC II$^+$ macrophages. **b**, Ly6C$^+$ iNOS$^+$ macrophages. **c**, Ly6C$^+$ CD206$^+$ macrophages. **d**, Ly6C$^+$ Arginase$^+$ macrophages. **e**, Ly6C$^+$ IL-10$^+$ macrophages. (a-e, n=8 mice per group, pooled from two independent experiments, two-tailed Mann–Whitney test). **a-e** Data are shown as the mean ± SD.
