# Supplementary material for: Chlamydia evasion of neutrophil host defense results in NLRP3 dependent myeloid-mediated sterile inflammation through the purinergic P2X7 receptor
Source: Nat Commun. 2021 Sep 15;12:5454. doi: 10.1038/s41467-021-25749-3 (PMC8443728; doi:10.1038/s41467-021-25749-3)
Supplement: Supplementary file 3 — Reporting summary [file 41467_2021_25749_MOESM3_ESM.pdf]

nature research

Corresponding author(s): Harlan Caldwell

Last updated by author(s): Aug 3, 2021

Reporting Summary

Nature Research wishes to improve the reproducibility of the work that we publish. This form provides structure for consistency and transparency in reporting. For further information on Nature Research policies, see our Editorial Policies and the Editorial Policy Checklist.

Statistics

For all statistical analyses, confirm that the following items are present in the figure legend, table legend, main text, or Methods section.

n/a	Confirmed	
☐	☒	The exact sample size (n) for each experimental group/condition, given as a discrete number and unit of measurement
☐	☒	A statement on whether measurements were taken from distinct samples or whether the same sample was measured repeatedly
☐	☒	The statistical test(s) used AND whether they are one- or two-sided<br>*Only common tests should be described solely by name; describe more complex techniques in the Methods section.*
☒	☐	A description of all covariates tested
☒	☐	A description of any assumptions or corrections, such as tests of normality and adjustment for multiple comparisons
☐	☒	A full description of the statistical parameters including central tendency (e.g. means) or other basic estimates (e.g. regression coefficient) AND variation (e.g. standard deviation) or associated estimates of uncertainty (e.g. confidence intervals)
☐	☒	For null hypothesis testing, the test statistic (e.g. F, t, r) with confidence intervals, effect sizes, degrees of freedom and P value noted<br>*Give P values as exact values whenever suitable.*
☒	☐	For Bayesian analysis, information on the choice of priors and Markov chain Monte Carlo settings
☒	☐	For hierarchical and complex designs, identification of the appropriate level for tests and full reporting of outcomes
☒	☐	Estimates of effect sizes (e.g. Cohen's d, Pearson's r), indicating how they were calculated

Our web collection on statistics for biologists contains articles on many of the points above.

Software and code

Policy information about availability of computer code

Data collection	No open-source or custom code was used to collect data for this paper.
Data analysis	GraphPad Prism 9 was used for graphical and statistical analyses. Flow Cytometry data was collected by FACSDiva 7.0 and analyzed by Flowjo v10. FASTX-Toolkit 0.0.14,Bowtie2 version 2.2.9 and GATK HaplotypeCaller version 4.1.2.0 were used for whole genome sequencing. RuneScape 2.5 was used for Ctr-16SrRNA probe hybridization. Linear Model for Micro Arrays (LIMMA) R package was used for proteomics study. Nikon Elements BR5.02.00 was used for fluorescence image processing and ImageJ Fiji was used for image analysis.

For manuscripts utilizing custom algorithms or software that are central to the research but not yet described in published literature, software must be made available to editors and reviewers. We strongly encourage code deposition in a community repository (e.g. GitHub). See the Nature Research guidelines for submitting code & software for further information.

Data

Policy information about availability of data

All manuscripts must include a data availability statement. This statement should provide the following information, where applicable:

- Accession codes, unique identifiers, or web links for publicly available datasets
- A list of figures that have associated raw data
- A description of any restrictions on data availability

The whole genome sequence data of CtD and CtD CT135- strains that support the findings of this study have been deposited with links to BioProject accession number PRJNA742023 in the NCBI BioProject database (https://www.ncbi.nlm.nih.gov/bioproject/). Genome sequence data are available in GenBank or the NCBI (accession no. NC_017437, NC_017435.1). The data that support the findings of this study are available from the corresponding author upon reasonable request.

Field-specific reporting

Please select the one below that is the best fit for your research. If you are not sure, read the appropriate sections before making your selection.

[x] Life sciences [ ] Behavioural & social sciences [ ] Ecological, evolutionary & environmental sciences

For a reference copy of the document with all sections, see nature.com/documents/nr-reporting-summary-flat.pdf

Life sciences study design

All studies must disclose on these points even when the disclosure is negative.

Sample size	The number of mice per group is standard or more for mouse experiments.
Data exclusions	No data were excluded from the present study.
Replication	5-10 chlamydia infected mice were performed in vivo study, the detailed mouse number was included in each figure legends. In vitro experiments including Chlamydia infected bone marrow derived neutrophil, bone marrow derived macrophage, McCoy, HeLa229 and BM12.4 cells with different treatment were successfully repeated two to three independent times.
Randomization	Mice used in the present study were randomly assigned to each group. Bone marrow derived neutrophil cells and bone marrow derived macrophage cells were purified from randomly assigned mice and divided into the different conditions for experiments. McCoy, HeLa229 and BM12.4 cells were cultured from the same condition and randomly divided into the different conditions for experiments.
Blinding	All animal were blindly coded for performing experiments. Data collection and in vitro experiment was performed by the same individual. Mouse infection was performed by different individuals. Data was decoded and analysis was performed by multiple investigators.

Reporting for specific materials, systems and methods

We require information from authors about some types of materials, experimental systems and methods used in many studies. Here, indicate whether each material, system or method listed is relevant to your study. If you are not sure if a list item applies to your research, read the appropriate section before selecting a response.

Materials & experimental systems

n/a	Involved in the study	
[ ]	[x]	Antibodies
[ ]	[x]	Eukaryotic cell lines
[x]	[ ]	Palaeontology and archaeology
[ ]	[x]	Animals and other organisms
[x]	[ ]	Human research participants
[x]	[ ]	Clinical data
[x]	[ ]	Dual use research of concern

Methods

n/a	Involved in the study	
[x]	[ ]	ChIP-seq
[ ]	[x]	Flow cytometry
[x]	[ ]	MRI-based neuroimaging

Antibodies

Antibodies used

Brilliant Violet 785 anti-CD45 (BioLegend, clone 30-F11,catalog number #103149);
Brilliant Violet 421 PE/Cy7 anti-CD11b (BioLegend, clone M1/70, catalog number #101236);
PE/Cy7 anti-F4/80 (BioLegend, clone BM8, catalog number #123114);
APC/Fire™ anti-Ly-6C (BioLegend, clone HK1.4, catalog number #128046);
FITC anti-I-A/I-E (BioLegend, clone M5/114.15.2, catalog number #107606);
FITC anti-CD206 (BioLegend, clone C068C2,catalog number #141704);
PerCP/Cyanine5.5 anti-CD80 (BioLegend,clone 16-10A1, catalog number #104722);
PE anti-IL-10 (BioLegend, clone JES5-16E3, catalog number #505008).
APC anti-TNF alpha (ThermoFisher Scientific, eBioscience™, clone MP6-XT22, catalog number #17-7321-82),
APC anti-Arginase 1 (ThermoFisher Scientific, eBioscience™, clone A1exF5, catalog number #17-3697-82)
PE-iNOS (ThermoFisher Scientific, eBioscience™, clone CXNFT, catalog number #47-5920-82)
Anti-Iba1 (FUJIFILM Wako, catalog number #019-19741)
Anti-Myeloperoxidase (Abcam, catalog number #ab9535)
Donkey anti-Rabbit IgG, Alexa Fluor 488 (ThermoFisher Scientific, Invitrogen™, catalog number #A21206)
Purified anti-mouse CD16/32 Antibody (BioLegend, clone 93, catalog number #101302)
RuneScape 2.5 LS Probe Ctr-16S rRNA (Advanced Cell Diagnostics, catalog number #462748)
Anti-chlamydial CT135 antibody was made by the Dr.Caldwell lab.
Anti-chlamydial IncA was provided by Dr. Ted Hackstadt.

Validation	Validation of all primary commercial antibodies for the species and application was warranted by the vendors. Validation statement can be found on the manufacturers' website. Anti-chlamydial IncA antibody was made and validated by Dr.Ted Hackstadt (Hackstadt T., et al. Cellular Microbiology (1999) 1(2), 119-130). Anti-chlamydial CT135 antibody was made and validated in this study.

Eukaryotic cell lines

Policy information about cell lines

Cell line source(s)	McCoy cell, HeLa 229 cell and BM12.4 cell.
Authentication	McCoy cell is purchased from ATCC (CRL-1696), HeLa229 cell was purchased from ATCC (CCL-2.1). BM12.4 cell was provided by Dr. Raymond Johnson from Yale University.
Mycoplasma contamination	Mycoplasma is tested by certified company, both BM12.4 cell and HeLa229 cell were Mycoplasma free.
Commonly misidentified lines (See ICLAC register)	No commonly misidentified cell lines were used in the study.

Animals and other organisms

Policy information about studies involving animals; ARRIVE guidelines recommended for reporting animal research

Laboratory animals	Six to eight-week-old female C57BL/6, Rag1-/- mice (line number 146), Rag2-/- mice (stock number: RAGN12-F), Rag2/Il2rg-/- (stock number: 4111-F) deficient in T, B and innate lymphoid cells (ILCs), Pfp/Rag2-/- (stock number: 1177) and TLR3-/- (line number 302) were obtained from Taconic Laboratories through a NIAID contract. NLRP3-/- (stock number: 021302), TNF p55-/-/p75-/- (TNFR1/2-/-; stock number: 003243), P2X7R-/- (stock number: 005576), AIM2-/- (stock number: 013144), Caspase1/11-/- (stock number: 016621), TLR2-/- (stock number: 004650), TLR4-/- (stock number: 007227), TLR7-/- (stock number: 008380), TLR9-/- (stock number: 34329), MyD88-/- (stock number: 009088), NOD2-/- (stock number: 005763), IL-1R-/- (stock number: 003245), TRIF-/- (stock number: 005037), STING-/- (stock number: 025805), cGAS-/- (stock number: 026554) mice were purchased from Jackson Laboratories. NLRC4-/- mice were provided by Dr. Gabriel Nunez (University of Michigan). Casp1-/- mice were provided by Dr. Thirumala-Devi Kanneganti (St. Jude Children's Research Hospital). IL-1alpha-/- mice were made by Dr. Yoichiro Iwakura (University of Tokyo) and provided by Sunny Shin (University of Pennsylvania). IL-1beta-/- IL-18-/- mice were made by Dr. David Chaplin (University of Alabama) and provided by Dr. Edward Miao (University of North Carolina). GBPchr3-/- mice was made by Drs. Masahiro Yamamoto and Kiyoshi Takeda and provided by Dr. Jorn Coers (University of Duke). NOD1-/- mice were provided by Dr. Dragana Jankovic (NIAID/NIH). Mice were maintained on 12 hours light/12 hours dark cycles under 18-23°C with 40-60% humidity at NIAID animal facility.
Wild animals	No wild animal was used in this study.
Field-collected samples	No field-collected samples were used in this study.
Ethics oversight	The animal research performed in this study was approved by the NIAID Animal Care and Used Committee.

Note that full information on the approval of the study protocol must also be provided in the manuscript.

Flow Cytometry

Plots

Confirm that:

- [x] The axis labels state the marker and fluorochrome used (e.g. CD4-FITC).
- [x] The axis scales are clearly visible. Include numbers along axes only for bottom left plot of group (a 'group' is an analysis of identical markers).
- [x] All plots are contour plots with outliers or pseudocolor plots.
- [x] A numerical value for number of cells or percentage (with statistics) is provided.

Methodology

Sample preparation	Mouse uterine horn were cut into small pieces, washed with HBSS containing 25 mM HEPES, 5 mM EDTA and 10% heat-inactivated horse serum (Life Technologies) at room temperature for 15 minutes and digested with RPMI 1640 containing 10% horse serum (Hyclone) with 1 mg/mL Collagenase D (Roche), 1 mg/mL Dispase II (Roche) and 0.25 mg/mL DNase I (Sigma) and shaken for 1 h at 37 °C. Cell suspensions were filtered through 70-µm nylon cell strainers.
Instrument	Becton Dickinson (BD) LSRII flow cytometer
Software	Flow Cytometry data was collected using FACSDiva 7.0 Software and analyzed using FlowJo v10.
Cell population abundance	Purity of the macrophage from mouse uterine horn were determined to be 55% or greater.

Gating strategy	Macrophages were gated as live, singlets, CD45(positive), CD11b(positive), F480(positive)

☒ Tick this box to confirm that a figure exemplifying the gating strategy is provided in the Supplementary Information.
